# The Interaction of Hemin, a Porphyrin Derivative, with the Purified Rat Brain 2-Oxoglutarate Carrier

**DOI:** 10.3390/biom11081175

**Published:** 2021-08-09

**Authors:** Daniela Valeria Miniero, Anna Spagnoletta, Nicola Gambacorta, Vito Scalera, Ciro Leonardo Pierri, Orazio Nicolotti, Annalisa De Palma

**Affiliations:** 1 Department of Biosciences, Biotechnologies, and Biopharmaceutics, University “Aldo Moro” of Bari, Via E. Orabona, 4, I-70125 Bari, Italy; vito.scalera@uniba.it (V.S.); ciro.pierri@uniba.it (C.L.P.); 2 ENEA Italian National Agency for New Technologies, Energy and Sustainable Economic Development, Trisaia Research Centre, S.S. 106 Jonica, Km 419,500, I-75026 Rotondella, Italy; anna.spagnoletta@enea.it; 3 Dipartimento di Farmacia—Scienze del Farmaco, Università degli Studi di Bari “Aldo Moro”, Via E. Orabona, 4, I-70125 Bari, Italy; nicola.gambacorta1@uniba.it (N.G.); orazio.nicolotti@uniba.it (O.N.); 4 BROWSer S.r.l. c/o, University “Aldo Moro” of Bari, Via E. Orabona, 4, I-70125 Bari, Italy

**Keywords:** mitochondrial carrier, kinetic study, inhibition, single binding centre gated pore mechanism, porphyrin derivatives, induced-fit molecular docking

## Abstract

The mitochondrial 2-oxoglutarate carrier (OGC), isolated and purified from rat brain mitochondria, was reconstituted into proteoliposomes to study the interaction with hemin, a porphyrin derivative, which may result from the breakdown of heme-containing proteins and plays a key role in several metabolic pathways. By kinetic approaches, on the basis of the single binding centre gated pore mechanism, we analyzed the effect of hemin on the transport rate of OGC in uptake and efflux experiments in proteoliposomes reconstituted in the presence of the substrate 2-oxoglutarate. Overall, our experimental data fit the hypothesis that hemin operates a competitive inhibition in the 0.5–10 µM concentration range. As a consequence of the OGC inhibition, the malate/aspartate shuttle might be impaired, causing an alteration of mitochondrial function. Hence, considering that the metabolism of porphyrins implies both cytoplasmic and mitochondrial processes, OGC may participate in the regulation of porphyrin derivatives availability and the related metabolic pathways that depend on them (such as oxidative phosphorylation and apoptosis). For the sake of clarity, a simplified model based on induced-fit molecular docking supported the in vitro transport assays findings that hemin was as good as 2-oxoglutarate to bind the carrier by engaging specific ionic hydrogen bond interactions with a number of key residues known for participating in the similarly located mitochondrial carrier substrate binding site.

## 1. Introduction

Heme is an endogenous porphyrin essential for several biological processes of aerobic cells, such as the transport and storage of oxygen, signal transduction mechanisms and structural components of hemeproteins. Heme-like derivatives play a crucial function in oxidative phosphorylation and oxygen consumption [1,2]. Biosynthesis of heme is a multistep process between the mitochondria and cytosol that involves condensation between glycine and succinyl-CoA to form 5-aminolevulinate in the mitochondria, catalyzed by δ-aminolevulinate synthase 2 (ALAS2) [3,4,5]. Following its synthesis, 5-aminolevulinate is transported to the cytosol where it leads to a series of reactions that culminate in the formation of co-protoporphyrinogen III [3,4,5]. Then, it re-enters the mitochondria where it is decarboxylated and oxidized to protoporphyrin IX, which incorporates ferrous iron by the reaction catalyzed by ferrochelatase to form heme [6,7,8,9]. Therefore, a tight regulation of the presence of its precursors and heme itself into the intermembrane space and into the mitochondrial matrix might be crucial for heme prosthetic group biosynthesis, as well as for the assembly of heme proteins.

Although all the enzymatic steps leading to the production of heme are well characterized [5,6,7,8,9], the mechanism of mitochondrial heme homeostasis is not yet clear. Transport of porphyrins in the isolated mitochondria is energy-dependent, as expected for the movement of anions into a negatively charged environment [10,11,12]. Protoporphyrin IX is slightly negatively charged at physiological pH and an anion of this size would not penetrate the inner mitochondrial membrane unless mediated by some mechanism(s) other than passive diffusion. Deuteroporphyrin IX [9] as well as protoporphyrin IX [11] have been shown to be accumulated in the mitochondria by a carrier-mediated transport-like mechanism.

Recently, some studies have highlighted the involvement of mitochondrial carriers in the accumulation of porphyrin derivatives into mitochondria [13,14]. Interestingly, the 2-oxoglutarate carrier (OGC) and the adenine nucleotide translocator (ANT), members of the mitochondrial carrier family located into the inner mitochondrial membrane [15,16], are able to bind some porphyrin derivatives with a crucial role in heme biosynthesis and their functions appear to be related to the accumulation of heme precursors into the mitochondrial matrix [13,14].

Among porphyrin derivatives playing a role in mitochondrial metabolism, hemin is one of the most studied molecules due to its ability in regulating mitochondrial function as a single molecule or in combination with other small molecules [17,18,19]. It was also reported that hemin may be released from hemoglobin as a consequence of central nervous system (CNS) hemorrhage and is present at high micromolar concentrations in intracranial hematomas [20]. Although hemin’s real protective/dangerous effects on mitochondrial function are still a matter of debate, it is known that free hemin in mammals can exert a neuroprotective effect with the expression of neuroglobin (Ngb) [21], and it also appears to be able to trigger or prevent mitochondrial dysfunction, depending on the targeted pathways in different tissues [17,18]. Hemin also shows regulatory functions such as the repression of nonspecific δ-aminolevulinate synthase expression [22], and can stimulate growth of oral bacteria, acting as an indicator of possible pathological conditions [23]. Hemin therapy has already been demonstrated to be effective in the treatment of heme-deficiency-related disorders such as porphyria [24]. It should be noticed that free hemin might also result from the breakdown of hemin-containing proteins highlighted in several microorganisms, such as *Porphyromonas gingivalis* [25] or *Bartonella henselae* [26].

At the mitochondrial level, it is known that hemin induces the activity of heme oxygenase, preventing alterations or reducing oxidative stress. It seems likely that the antioxidant effects associated with heme oxygenase upregulation are responsible for both the protective effect of hemin administration on mitochondrial function and its action to prevent protein oxidation/lipid peroxidation [17,18].

Given the role played by hemin in regulating redox homeostasis, the ability of OGC in binding heme-like molecules [27] and the involvement of OGC in maintaining redox homeostasis through its participation in the malate/aspartate shuttle [28], we try to ascertain if OGC can bind hemin.

As a mitochondrial transporter, OGC is an antiporter and transports through the mitochondrial inner membrane the 2-oxoglutarate coming from the cytosol into mitochondria via an electroneutral exchange for malate from the mitochondrial matrix [29,30,31,32]. OGC is very important for several metabolic reactions, including the citric acid cycle, gluconeogenesis and nitrogen metabolism, beyond the transfer of reducing equivalents through the malate/aspartate shuttle [33,34].

From a structural point of view, OGC, as a member of mitochondrial carriers, shows a tripartite structure, thus consisting of three tandem-repeated sequences of about 100 amino acids in length [29,30,31,32,33,34]. Each repeat contains two hydrophobic stretches that span the inner mitochondrial membrane as α-helices and a characteristic sequence motif consisting of residues P-X-D/E-XX-K/R-(20–30 residues)-EG-XXXXX-Aromatic residue-K/R-G linking the two helices of each repeat, entirely conserved in all the members of the mitochondrial carrier family, also through phylogenetically distant species [35,36,37,38,39,40,41,42].

The OGC carrier has been studied by site-directed mutagenesis [43,44,45]; it has been purified from various sources [46] and the characterization of the transport mechanism has been reported [30,31,32,47]. The OGC from rat brain mitochondria, reconstituted into proteoliposomes, was studied by kinetic approaches, reaching the conclusion that the carrier can swap its substrates according to the single binding centre gated pore mechanism. Based on this mechanism, a single binding site is alternatively accessible from the matrix or the cytosol, as demonstrated for the ADP/ATP carrier, tricarboxylate carrier and the OGC, modifying the employed equations for the presence of a further ligand [16,48,49]. Herein, we have examined the influence of a porphyrin derivative, namely hemin, on the transport of labeled substrates mediated by the reconstituted OGC, isolated from rat brain mitochondria in order to gain more direct information about its ability to bind OGC and/or behave as a modulator of OGC function.

## 2. Materials and Methods

### 2.1. Chemicals

In regards to chemicals: [^14^C] 2-oxoglutarate and [^14^C] malate were purchased from Perkin-Elmer Life Sciences; hydroxylapatite (Bio-gel HTP) and Amberlite Bio-Beads SM-2 from Bio-Rad; Triton X-100, Triton X-114, acrylamide and *N*,*N*′-methylenebisacrylamide from Serva; egg yolk phospholipids from Fluka; Matrex Gel Orange from Amicon (Beverly, MA, USA); hemin, cardiolipin, 1,4-piperazine-diethanesulphonicacid (Pipes), sodium dodecyl sulfate (SDS) and asolectin from Sigma; celite 535 from Roth and Sephadex G-75 from Pharmacia. All other chemicals used were of analytical grade.

### 2.2. Purification of the OGC Carrier

The 2-oxoglutarate carrier was purified from rat brain mitochondria according to a protocol described in [48]. Briefly, mitochondria were solubilized with a solution containing 3% Triton X-100 (*w*/*v*), 20 mM Na_2_SO_4_, 1 mM EDTA and 10 mM Pipes, pH 7.0 at a final protein concentration of 10 mg/mL. After the incubation at 4 °C for 10 min, the mixture was supplemented with 4 mg/mL of cardiolipin and centrifuged at 15,000× *g* for 15 min. After, the supernatant was applied to about 0.6 g of cold hydroxylapatite/celite (5:1) columns and eluted in the presence of 3% Triton X-100 (*w*/*v*). The first 0.6 mL fraction was collected and applied on a cold Matrex Gel Orange column pre-equilibrated with 4 mL of 0.1% Triton X-100 (*w*/*v*) in the presence of 10 mM Na_2_SO_4_, 1 mM EDTA and 5 mM Pipes, pH 7.0 with added 2 mg/mL asolectin. Fractions of 0.8 mL were collected and pure OGC was present in the second fraction with an apparent molecular weight of 35 kDa [48]. All the operations were performed at 4 °C.

### 2.3. Reconstitution of the OGC into Liposomes

Purified OGC was reconstituted into liposomes by removing the detergent using a micro-batchwise method in the presence of ion-exchange resin Bio-Beads SM-2 as described in ref. [50]. The initial mixture used for reconstitution was constituted by 200 μL of the purified OGC, 100 μL of 10% (*w*/*v*) Triton X-114, 80 μL of 10% (*w*/*v*) egg yolk phospholipids in the form of sonicated liposomes, 2-oxoglutarate, 200 μL of 10 mg/mL asolectin and 10 mM Pipes, pH 7.0, in a final volume of 700 μL. After vortexing, the mixture was transferred into the Eppendorf tube (2 mL) containing 0.4 g Amberlite Bio-Beads SM-2 and, after rotating at 32 rpm, the proteoliposomes were recovered by gentle aspiration. All the operations were performed at room temperature.

### 2.4. Transport Measurements

The external substrate was removed by passing 650 μL of the proteoliposomes through a Sephadex G-75 column (0.7 × 15 cm), pre-equilibrated with 50 mM NaCl/10 mM Pipes (pH 7.0). The first turbid fraction from the Sephadex G-75 column (600 μL) was collected and distributed into reaction vessels (100 μL each) for transport measurements by the inhibitor stop method [51]. The transport activity was determined by measuring the flux of labeled 2-oxoglutarate from inside to outside (efflux experiments) or outside to inside (uptake experiments). For efflux experiments, proteoliposomes containing 6 mM internal 2-oxoglutarate were loaded with 0.1 mM [^14^C] 2-oxoglutarate at high specific radioactivity for 30 min at 25 °C by carrier-mediated exchange equilibrium. After incubation time, the external radioactivity was removed by passing the proteoliposomes through Sephadex G-75 columns pre-equilibrated with 50 mM NaCl. Transport was started by adding either unlabeled 2-oxoglutarate at the indicated concentrations in a buffer of 50 mM NaCl, 10 mM Pipes (pH 7.0) (backward exchange), buffer alone, DMSO solvent or hemin, as indicated in the context of each experiment and stopped at the desired time. In the case of uptake experiments (forward exchange), transport was started by adding from outside the labeled substrate to proteoliposomes containing 2-oxoglutarate and stopped after 2 min by adding 10 μL of 350 mM pyridoxal 5′-phosphate (PLP) as an inhibitor. External radioactivity was removed by a Sephadex G-75 column (0.6 × 8 cm) and the proteoliposomes eluted with 1.2 mL of 50 mM NaCl were collected in 4 mL of scintillation mixture. In control samples, the inhibitor was added together with the labeled substrate at time zero and run in duplicate with the assay temperature at 25 °C. The transport activity was calculated by subtracting the control value from the experimental values, and expressed as mmol/g protein or as intraliposomal counts per minute (cpm). The values represented are the means ± SD from three independent experiments.

### 2.5. Protein Quantification

Polyacrylamide gel electrophoresis of precipitated proteins was performed in accordance to the method of Laemmli in the presence of 0.1% SDS [52]. The stacking gel (5% *w*/*v* of polyacrylamide) and the separation gel (17.5% *w*/*v* of polyacrylamide) were prepared with an acrylamide/bisacrylamide ratio of 38:1. Staining was performed by the silver nitrate method [53] and protein concentration was determined by the Lowry method modified for the presence of non-ionic detergents [54]. All the samples used for protein determination were dissolved in 1% (*w*/*v*) SDS.

### 2.6. Induced-Fit Molecular Docking

The homology model of the OGC carrier 3D structure herein used was built with Modeller [55] by taking the structure of the bovine AAC1 as a template, whose entry in the Protein Data Bank is 1OKC [56]. WHAT IF web server [57] was employed for assessing the goodness of both structural and energetic properties of the generated 3D comparative model. More details are available from the reference papers [58,59]. The OGC 3D structure was subsequently treated with the Protein Preparation Tool [60] of the Schrodinger suite for further checks on protonation states at physiological pH and tautomers. Hemin structure was retrieved from the Cambridge Crystallographic Data Centre (CCDC) [61,62]. A zero-order bond was assigned for flagging coordination bonds between Fe^3+^ cations and nitrogen atoms of the porphyrin ring and between Fe^3+^ cations and Cl^−^ anions. The 2-oxoglutarate structure was treated with the LigPrep tool [60] in order to generate all possible tautomerization states along with protonation states at physiological pH. Ligand structures were finally minimized by employing prime package [63]. The induced-fit docking protocol [63] was employed by using Glide [64] with an OPLS3e force field [65] in order to inspect the binding mode of the selected ligands together with conformational changes within the receptor, the latter not detectable in the standard docking protocol. Notably, the employed induced-fit docking protocol is coherent with the induced transition fit of transport catalysis, commonly accepted for explaining substrate translocation along mitochondrial carriers [66]. The enclosing box was centered on the center of mass of key residues R288, R190 and R90, representing the crucial residues of the OGC binding catalytic pocket when OGC is open towards the cytosolic side, according to the similarly located shared mitochondrial binding site [36]. Side-chain predictions were performed on residues within 6 Å from ligand poses together with Glide SP redocking of each protein–ligand complex structure within 30.0 kcal/mol of the lowest energy.

## 3. Results

**Figure 1:**
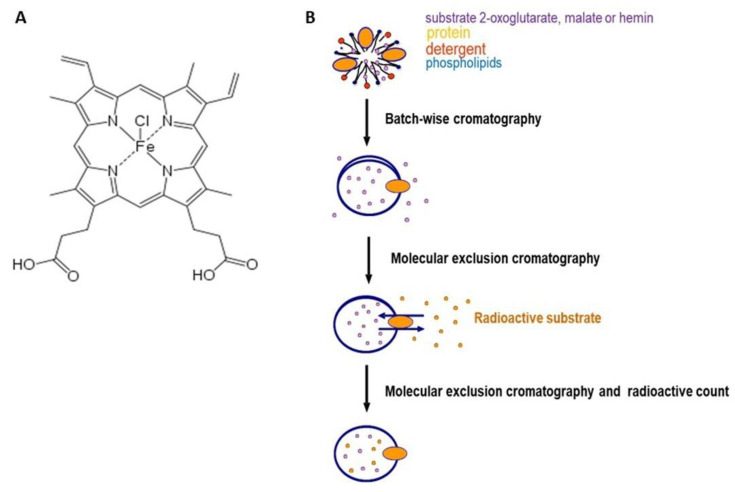
Panel (**A**), chemical structure of hemin. Panel (**B**), schematic representation of protein reconstitution and transport system.

The effect of hemin (Figure 1, panel A), a porphyrin derivative, on the uptake of labeled 2-oxoglutarate or labeled malate mediated by the purified OGC (Figure 1, panel B) was investigated.

**Figure 2:**
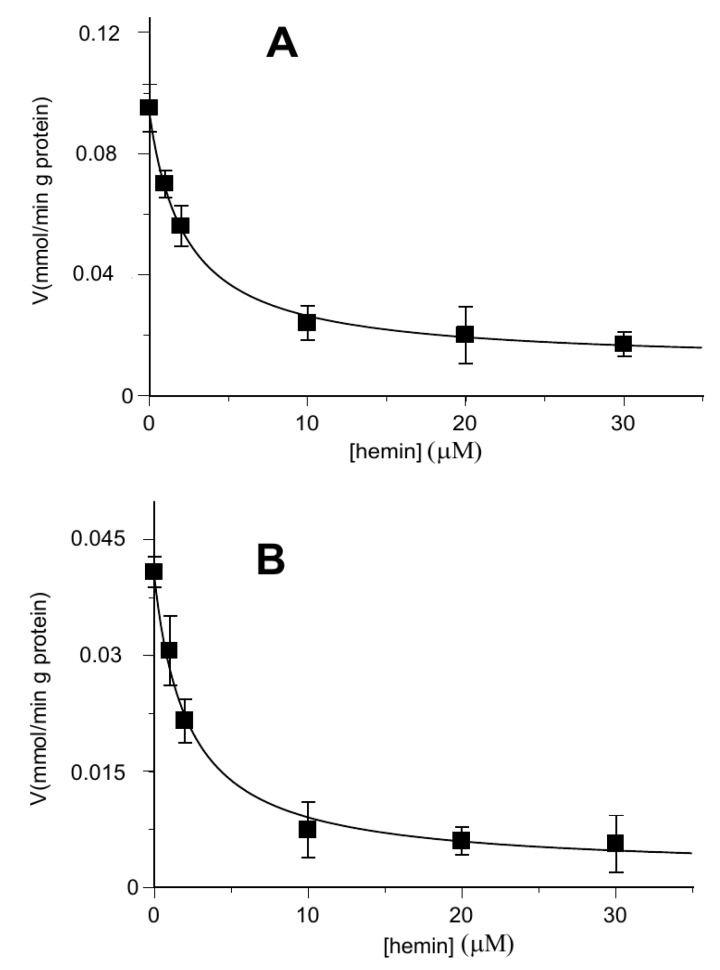
Hemin inhibition of the OGC-mediated uptake of [^14^C] 2-oxoglutarate and [^14^C] malate. The transport rate of [^14^C] 2-oxoglutarate in homo-exchange (**A**) or [^14^C] malate in hetero-exchange (**B**) was measured in 2 min in the absence and in the presence of increasing concentrations of hemin (1–30 µM). Hemin was added together with [^14^C] 2-oxoglutarate or [^14^C] malate at a concentration of 0.05 mM to proteoliposomes containing 6 mM of 2-oxoglutarate. Values are means ± S.D. from three independent experiments.

Figure 2 shows that hemin exerted about 50% of inhibition on the [^14^C] 2-oxoglutarate or [^14^C] malate uptake rate at about 2 μM (IC_50_ 1.58 ± 0.22 μM and IC_50_ 1.66 ± 0.14 μM, respectively) in proteoliposomes loaded with internal 6 mM of 2-oxoglutarate.

**Figure 3:**
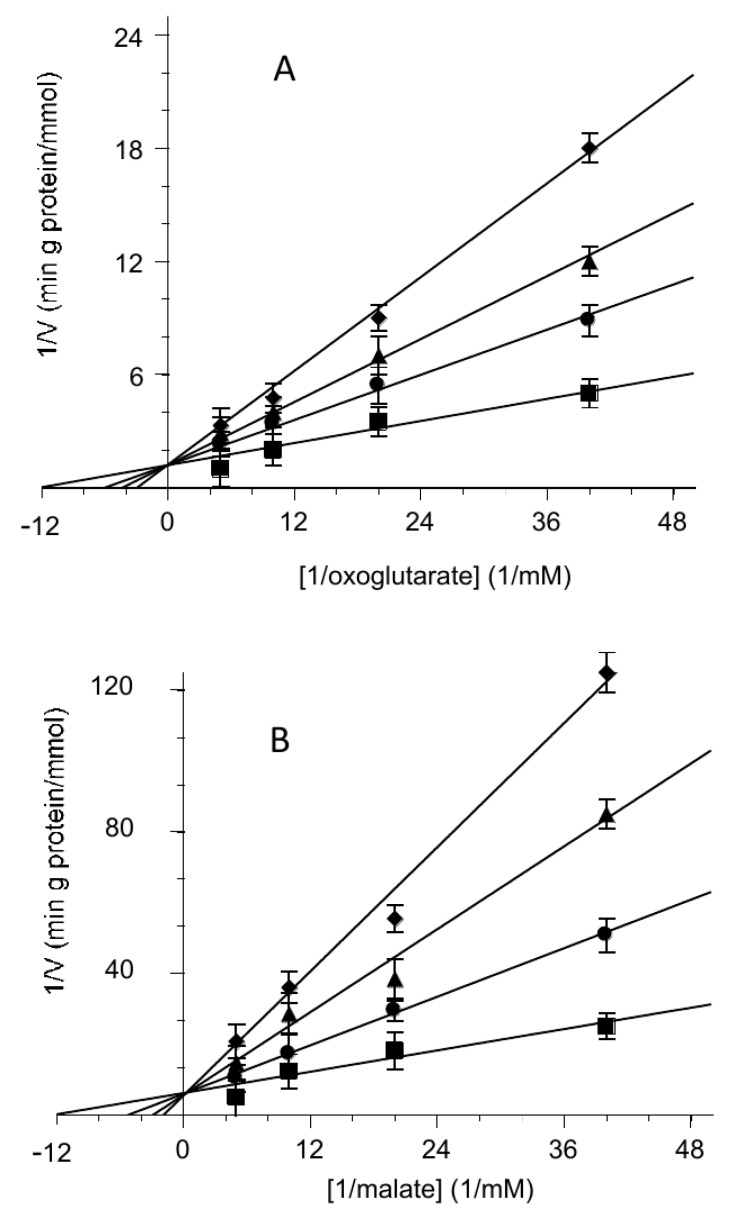
Competitive inhibition of the 2-oxoglutarate or malate transport activity by hemin. Double reciprocal plot showing the dependence of the uptake rate on hemin external concentrations. The transport rate of [^14^C] 2-oxoglutarate (**A**) or [^14^C] malate (**B**) in proteoliposomes containing 6 mM of 2-oxoglutarate was measured in 2 min in the absence (■) or presence of hemin at 0.5 µM (●), 2 µM (▲) and 3.5µM (♦). Hemin was added together with [^14^C] 2-oxoglutarate (**A**) or [^14^C] malate (**B**) at concentrations of 0.025 mM, 0.05 mM, 0.1 mM and 0.2 mM. The data are the means ± SD from three different experiments.

When the rate of uptake was measured as a function of the external substrate in the presence of different hemin concentrations, the inhibition was clearly revealed to be a competitive type both for the 2-oxoglutarate and the malate, as reported on double reciprocal plots (Lineweaver–Burk) of Figure 3A,B, respectively.

An obvious consequence of this result has been to verify whether hemin was a substrate of the OGC according to the single binding centre gated pore mechanism which implies that the carrier has a single binding site for its substrates and can rearrange itself between the inside and outside form, in both the free and substrate-bound states [16,48,49].

**Figure 4:**
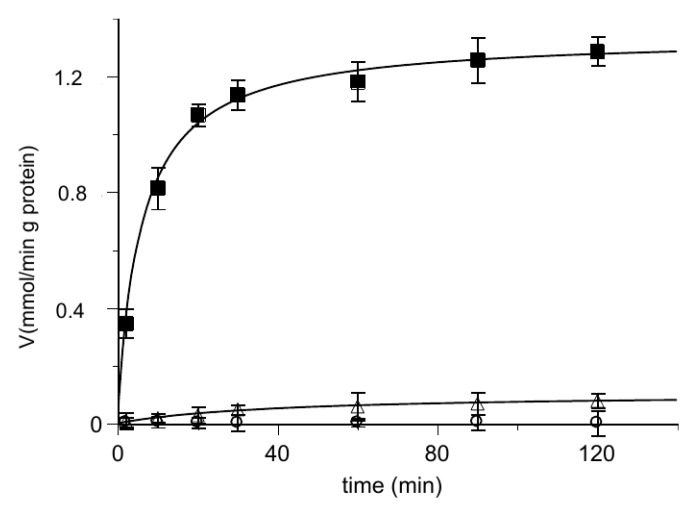
Time course of [^14^C] 2-oxoglutarate uptake in reconstituted proteoliposomes. At zero time, 0.025 mM [^14^C] 2-oxoglutarate is added to reconstituted proteoliposomes containing 6 mM of 2-oxoglutarate (■), 50 µM hemin (◯) or 10 mM Pipes (△). Transport was stopped by adding 10 μL of PLP 350 mM at the indicated times and the intraliposomal radioactivity was measured. The data are the means ± SD from three different experiments.

Then, we performed the uptake of [^14^C] 2-oxoglutarate into proteoliposomes loaded with 2-oxoglutarate or hemin, respectively. The results are described in Figure 4 where the time course of the labeled 2-oxoglutarate uptake was analyzed. The uptake in the presence of hemin inside the proteoliposomes was quite small with respect to the oxoglutarate/oxoglutarate exchange, at the same level of the very poor substrate uptake by proteoliposomes containing only the Pipes buffer. This rules out the possibility for hemin to be transported.

**Figure 5:**
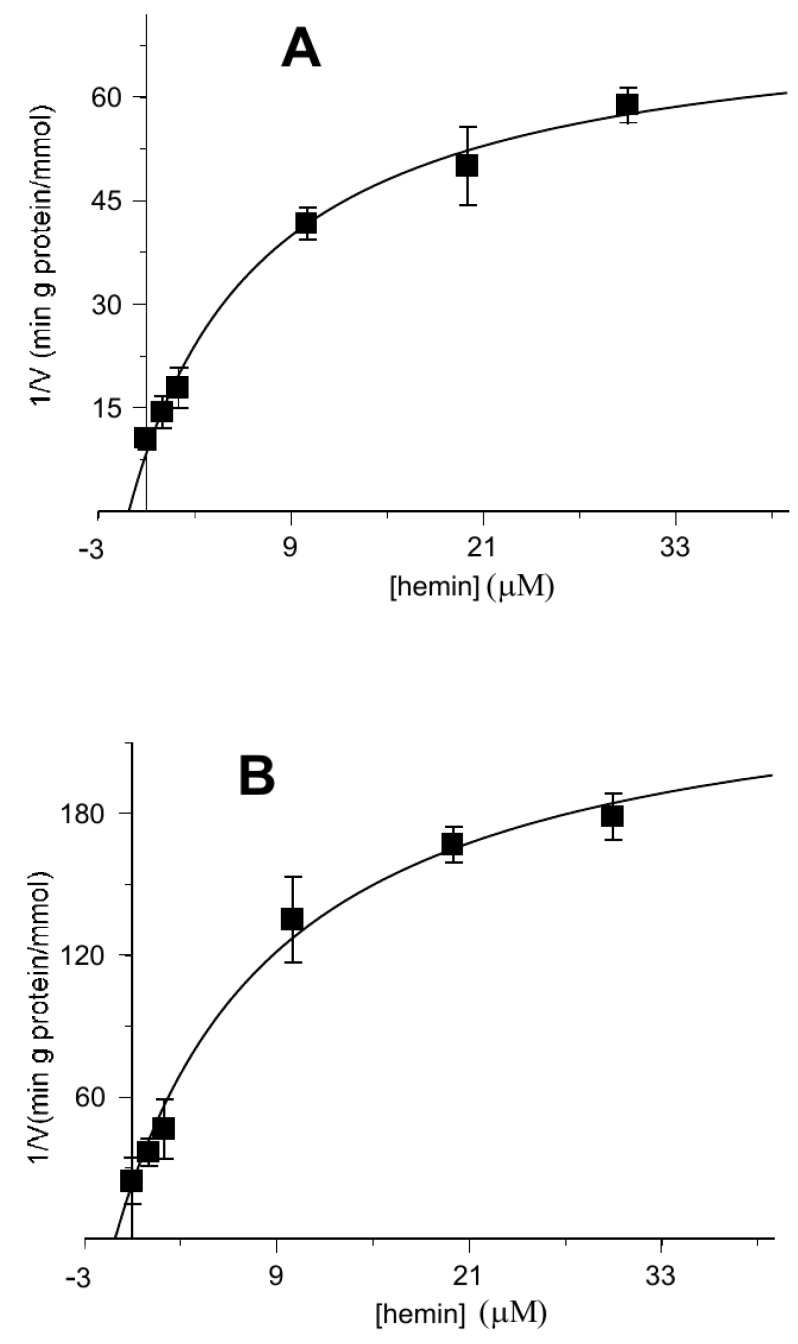
Kinetic analysis of the inhibition data of Figure 2 of the reconstituted oxoglutarate/oxoglutarate and malate/oxoglutarate exchanges by hemin using the Dixon plot. Hemin was added together with 0.05 mM of [^14^C] 2-oxoglutarate (**A**) or 0.05 mM [^14^C] malate (**B**) to proteoliposomes containing 6 mM of 2-oxoglutarate in the absence or in the presence of increasing hemin concentrations (1–30 µM).

Further information has been obtained by evaluating the uptake rate as a function of the hemin concentration, in a range indicated by the data of Figure 2. In the Dixon plots (Figure 5), the reciprocals of uptake rates for each substrate (2-oxoglutarate and malate) were plotted as a function of hemin concentrations. It is evident that in both cases the data could hardly fit to straight lines, as one would expect for a pure competitive inhibition in analogy to classic enzyme kinetics, as hemin concentration increases beyond 10 μM. Maybe it is due to the targeting of other protein regions not directly involved in substrate binding and/or translocation but likely involved in protein conformational changes (see also the Discussion Section). In addition, higher concentrations of hemin with phospholipids or additional kinetic regulatory effects might alter proteoliposome integrity, accordingly to what has been previously reported [67].

**Figure 6:**
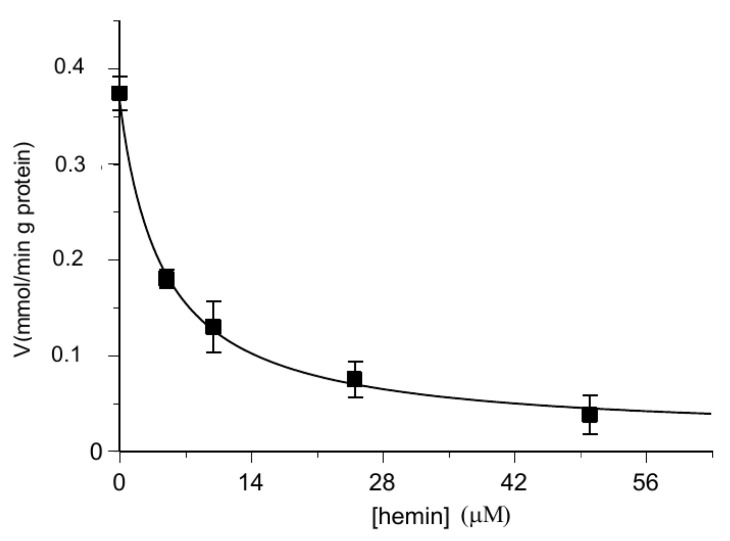
Inhibition of the transport activity of the [^14^C] 2-oxoglutarate in the OGC carrier by hemin preloaded within proteoliposomes. The transport rate of [^14^C] 2-oxoglutarate in proteoliposomes containing 6 mM of 2-oxoglutarate and increasing concentrations of hemin (0–50 μM) was measured in 2 min. Additionally, [^14^C] 2-oxoglutarate was added at a concentration of 0.025 mM. The values are the means ± SD from three independent experiments.

In order to verify if hemin inside the proteoliposomes behaved in the same way as outside them, experiments were performed with proteoliposomes loaded with hemin and substrate, then labeled 2-oxoglutarate uptake was analyzed. Once established that hemin alone had no effect (Figure 4), the experiment described in Figure 6 shows that, also in this case, hemin inhibited the substrate influx, again in the order of micromolar concentrations. The determined IC_50_ value of 1.99 ± 0.16 μM obtained by preloading proteoliposomes with hemin (Figure 6) is comparable to those obtained from the experiments in which hemin was added from the outside (Figure 2).

**Figure 7:**
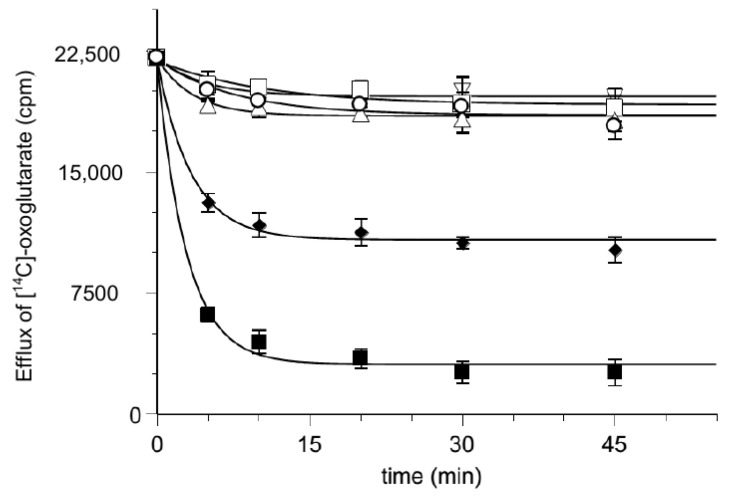
Effect of hemin on the time course of [^14^C] 2-oxoglutarate efflux from proteoliposomes. Efflux of [^14^C] 2-oxoglutarate from pre-labeled proteoliposomes with radioactive substrates was measured as described in Materials and Methods in the presence of external 50 mM NaCl and 10 mM Pipes pH 7 buffer (▽), DMSO solvent (△), 0.025 mM 2-oxoglutarate (♦), 0.25 mM 2-oxoglutarate (■), 0.010 mM hemin (☐) and 0.010 mM hemin with 0.025 mM 2-oxoglutarate (◯) added at time 0. The data are the means ± SD from three different experiments.

Furthermore, loading proteoliposomes with labeled 2-oxoglutarate makes it possible to analyze the substrate efflux step occurring during the exchange process, both in the presence and the absence of hemin (Figure 7). Additionally, in this case the possibility for hemin to be transported is excluded.

### Molecular Docking Analyses

**Figure 8:**
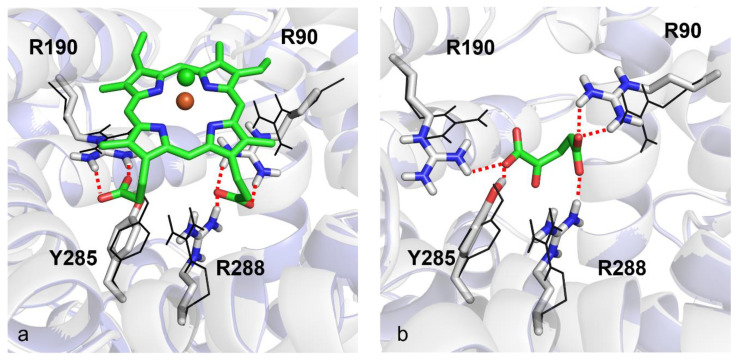
Induced-fit molecular docking. Panels (**a**) and (**b**) show the top-scored poses returned from docking analyses for hemin and 2-oxoglutarate within the OGC homology model built by using the bovine crystallized AAC1 as a template (PDB_ID: 1OKC). Red dotted lines indicate hydrogen bonds. Fe^3+^ and Cl^−^ ions are depicted as orange and green spheres, respectively. Black wireframes show the initial positions of key residue side-chains before induced-fit docking simulations.

For investigating the putative interaction of hemin with the OGC binding region proposed for 2-oxoglutarate, molecular docking analyses were performed to shed light on the interactions of hemin and 2-oxoglutarate towards OGC, whose 3D structure was built by homology modeling based on the bovine AAC1 template retrieved from the Protein Data Bank with code 1OKC. As shown in Figure 8, a network of electrostatic and hydrogen bond interactions is shared between hemin and 2-oxoglutarate. In particular, the carboxylic groups of 2-oxoglutarate can interact with R288, R90 and R190 as well as with the side-chain of Y285. On the other hand, the carboxyl groups on the hemin arms can play the same ionic interactions experienced from 2-oxoglutarate with protonated nitrogen atoms of R90, R190 and R288 residues. Notably, the three residues represent the main three contact points of the mitochondrial carrier similarly located on the common substrate binding site [37,68]. For the sake of completeness, docking score values returned from in silico analyses were equal to −10.182 kcal/mol and −5.519 kcal/mol for hemin and 2-oxoglutarate, respectively, making these results coherent with the experimental data.

## 4. Discussion

The general equation of the rate of substrate transport reported in refs. [48,49] describes the rate of influx of a substrate into the internal phase of proteoliposomes (or any space delimited by a membrane), when mediated by a specific carrier-protein according to the single binding centre gated pore mechanism [16,48,49]. In analogy with enzyme kinetics, we use the reasonable postulate that the interactions between the carrier and its substrates represent the fast steps of the process. The rearrangement steps of the carrier inside the membrane, leading to the exposition of the binding site (either with a bound substrate or free) towards the intermembrane space (carrier in the cytosolic c-conformation, refs. [36,43,44,45,46,56]) or towards the matrix space (carrier in the matrix m-conformation) [69,70,71], are relatively slow and thus rate determining. Consequently, in any transport rate equation, the rate “v” will depend on the concentration of the substrates and inhibitors, as well as on their dissociation constants and on the kinetic constants of the rearrangement steps. In particular, we use the following notations:

A = (2-oxoglutarate) B = (L-malate) E = (hemin),

K_A_ = Carrier–2-oxoglutarate dissociation constant,

K_B_ = Carrier–L-malate dissociation constant,

K_E_ = Carrier–hemin dissociation constant,

k_2_ and k_–2_ = kinetic constants of outside–inside and inside–outside rearrangement of the carrier–oxoglutarate complex,

k_3_ and k_−3_ = kinetic constants of outside–inside and inside–outside rearrangement of the free carrier,

In the simple case of homo-exchange, with one substrate present in both the external A_e_ and the internal phase A_i_, the equation is:

(1) $${v=V}_{M}\cdot \frac{A_{e}}{A_{e}+K_{M}}$$

(2) $${\mathrm{where}\;V}_{M}=k_{2\;}\cdot C_{T}\cdot \frac{k_{-2}A_{i}+k_{-3}K_{\mathrm{Ai}}}{\left(k_{-2}+k_{2}\right)A_{i}+\left(k_{-3}+k_{2}\right)K_{\mathrm{Ai}}}$$

(3) $${\;\mathrm{and}\;K}_{M}=\frac{\left(k_{-2}+k_{3}\right)A_{i}+\left(k_{-3}+k_{3}\right)K_{\mathrm{Ai}}}{\left(k_{-2}+k_{2}\right)A_{i}+\left(k_{-3}+k_{2}\right)K_{\mathrm{Ai}}}K_{\mathrm{Ae}}$$

when the external substrate is malate, the terms A_e_, K_Ae_ and k_2_ are substituted by B_e_, K_Be_ and k_4,_ whereas when malate is the internal substrate, the terms A_i_, K_Ai_ and k_−2_ are substituted by B_i_, K_Bi_ and k_−4_.

The presence of a third molecule (hemin) outside the proteoliposomes, able to interact with the carrier, requires a new elaboration of the rate equation, taking this presence into account.

In fact, hemin is revealed to be a very effective inhibitor of the oxoglutarate/oxoglutarate and malate/oxoglutarate exchange promoted by the OGC carrier purified from rat brain mitochondria and reconstituted into proteoliposomes.

According to our transport assays, hemin competitively inhibits the transport of both substrates, 2-oxoglutarate and malate (Figure 3A,B), and this could be easily explained by an interaction between hemin and the OGC while competing with the substrate. For analysing transport assays in the presence of hemin, the rate equation would contain two additional terms: the hemin concentration and the dissociation constant of the hemin–carrier complex. The modified equation again has the Michaelian saturation form as in Equation (1), with an unmodified V_M_ and a K_M_ that is a linear function of the hemin concentration; as for the competitive inhibition in enzyme kinetics:

(4) $$K_{M}=\frac{\left(k_{-2}A_{i}+k_{-3}K_{\mathrm{Ai}}\right)E+\left(\left(k_{-2}+k_{3}\right)A_{i}+\left(k_{-3}+k_{3}\right)K_{\mathrm{Ai}}\right)K_{E}}{\left(\left(k_{-2}+k_{2}\right)A_{i}+\left(k_{-3}+k_{2}\right)K_{\mathrm{Ai}}\right)K_{E}}K_{\mathrm{Ae}}$$

In addition, the equations for the rate “v” and its reciprocal “1/v” as functions of the inhibitor concentration can be derived:

(5) $$v=\frac{V_{0}}{1+\frac{1}{K_{0.5}}E}$$

(6) $$\mathrm{and}\;\frac{1}{v}=\frac{1}{V_{0}}+\frac{1}{V}\cdot \frac{K_{\mathrm{Ae}}}{K_{E}}\cdot E$$

with

(7) $$V_{0}=V\cdot \frac{\left(k_{-2}A_{i}+k_{-3}K_{\mathrm{Ai}}\right)A_{e}}{\left(\left(k_{-2}+k_{2}\right)A_{i}+\left(k_{-3}+k_{2}\right)K_{\mathrm{Ai}}\right)A_{e}+\left(\left(k_{-2}+k_{3}\right)A_{i}+\left(k_{-3}+k_{3}\right)K_{\mathrm{Ai}}\right)K_{\mathrm{Ae}}}$$

And

(8) $$K_{0.5}=\frac{\left(\left(k_{-2}+k_{2}\right)A_{i}+\left(k_{-3}+k_{2}\right)K_{\mathrm{Ai}}\right)A_{e}+\left(\left(k_{-2}+k_{3}\right)A_{i}+\left(k_{-3}+k_{3}\right)K_{\mathrm{Ai}}\right)K_{\mathrm{Ae}}}{\begin{array}{c} \left(k_{-2}A_{i}+k_{-3}K_{\mathrm{Ai}}\right)K_{\mathrm{Ae}}\\ \cdot \end{array}}K_{\mathrm{Ee}}$$

where V_0_ and K_0.5_ depend on A_e_ with A_i_ constant.

The rate “v” is a decreasing asymptotic function of “E”, with the abscissa as an asymptote at any substrate concentration, while “1/v” is a linear function of the inhibitor (Dixon plot). Our experimental data of Figure 2 and 6 fit to these decrement trends. In Figure 5 the linear trend is evident below the point at 10 μM of hemin, which can bind OGC by inhibiting 2-oxoglutarate and malate translocation. At higher concentrations the straight line curves, probably due to damage of proteoliposomes as supported by previous observations, showing that high concentrations of hemin and porphyrin-like derivatives destabilize biological membranes and liposomes [67].

Finally, for supporting the hypothesis formulated about the role played by hemin as an OGC inhibitor, molecular docking analyses showed that the negatively charged carboxylic groups of hemin interact with the three arginine residues, forming the three contact points of the OGC substrate binding region with an orientation very similar to the orientation observed for the carboxylic functional groups of 2-oxoglutarate, when the carrier is in c-conformation.

Moreover, hydrogen bonds with R288, R190 and R90 stabilize the structure of hemin within the OGC binding pocket, forming a protein–ligand complex even stronger than the OGC–2-oxoglutarate protein–ligand complex (OGC–hemin docking score equal to −10.182 kcal/mol versus OGC–2-oxoglutarate docking score equal to −5.519 kcal/mol).

Although hemin can be detected in various body fluids such as saliva and urine under various pathological states, it exists in cells at a very low concentration (μM range). Some previous papers state that about 80% of total hemin in plasma binds initially to LDL and HDL, while in mitochondria hemin might be present at concentration < 10 μM [72,73]. It is known that OGC participates in the malate/aspartate shuttle used by mammalia cells as a pathway for transferring reducing equivalents from mitochondria to the cytosol [28]. The impairment of the malate/aspartate shuttle may trigger the impairment of mitochondrial respiration and thus inhibitory interactions between hemin-like molecules and OGC may participate in regulating mitochondrial apoptosis. In addition, since hemin also appears to be able to trigger or prevent mitochondrial dysfunction, refs. [17,18] and free hemin in mammals can exert a neuroprotective effect [21], conversely it can induce mitochondrial dysfunction in endothelial cells and in neuroblastoma SH-SY5Y cells [17,74]. In light of our findings showing that OGC can interact with hemin on mitochondrial function [17,18], it might be speculated that OGC can bind hemin and/or porphyrin like ligands (i.e., released by heme-dependent protein complexes, partially damaged from oxidative stress, ageing or disease conditions). It is resulting in OGC inhibition and in the impairment of the malate/aspartate shuttle for favoring mitophagy/autophagy, in presence of a low excess of porphyrin-like molecules [17,18,19,20,21]. If the oxidative damage is more serious, in presence of an excess of hemin and other porphyrin-like molecules, the inhibition of malate/aspartate shuttle is more severe, favoring the alteration of lipid peroxidation pathways and mitochondrial apoptosis. Notably, mitochondrial apoptosis might also be favored by membrane leakage induced by the higher concentration of porphyrin-like molecules [19].

## Data Availability

Not applicable.
